# Evaluation of Abdominal Fetal Electrocardiography in Early Intrauterine Growth Restriction

**DOI:** 10.3389/fphys.2017.00437

**Published:** 2017-06-26

**Authors:** Clarissa L. Velayo, Kiyoe Funamoto, Joyceline Noemi I. Silao, Yoshitaka Kimura, Kypros Nicolaides

**Affiliations:** ^1^Department of Physiology, College of Medicine, University of the PhilippinesManila, Philippines; ^2^Department of Obstetrics and Gynecology, Graduate School of Medicine, Tohoku UniversitySendai, Japan; ^3^Department of Obstetrics and Gynecology, Philippine General Hospital, University of the PhilippinesManila, Philippines; ^4^Harris Birthright Research Centre, Kings College HospitalLondon, United Kingdom

**Keywords:** fetal electrocardiography, intrauterine growth restriction, prenatal screening, fetal cardiophysiology, fetal monitoring

## Abstract

**Objectives:** This descriptive study was performed to evaluate the capability of a non-invasive transabdominal electrocardiographic system to extract clear fetal electrocardiographic (FECG) measurements from intrauterine growth restricted (IUGR) fetuses and to assess whether abdominal FECG parameters can be developed as markers for evaluating the fetal cardiac status in IUGR.

**Methods:** Transabdominal FECG was attempted in 20 controls and 15 IUGR singleton pregnancies at 20+0−33+6 weeks gestation. Standard ECG parameters were compared between the study groups and evaluated for their correlation. Accuracy for the prediction of IUGR by cut off values of the different FECG parameters was also determined.

**Results:** Clear P-QRST complexes were recognized in all cases. In the IUGR fetuses, the QT and QTc intervals were significantly prolonged (*p* = 0.017 and *p* = 0.002, respectively). There was no correlation between ECG parameters and Doppler or other indices to predict IUGR. The generation of cut off values for detecting IUGR showed increasing sensitivities but decreasing specificities with the prolongation of ECG parameters.

**Conclusion:** The study of fetal electrocardiophysiology is now feasible through a non-invasive transabdominal route. This study confirms the potential of FECG as a clinical screening tool to aid diagnosis and management of fetuses after key limitations are addressed. In the case of IUGR, both QT and QTc intervals were significantly prolonged and thus validate earlier study findings where both these parameters were found to be markers of diastolic dysfunction. This research is a useful prelude to a test of accuracy and Receiver Operating Characteristics (ROC) study.

## Introduction

Intrauterine growth restriction (IUGR) affects 3–10% of all pregnancies and has been associated with the development of adult cardiovascular diseases.(Barker et al., 1989; Bahtiyar and Copel, 2008) Recent studies suggest that cardiovascular changes may be present during the prenatal period with evidence of cardiac remodeling in the form of increased transverse diameters and more globular cardiac ventricles (Crispi et al., 2010). These corroborate with earlier descriptions of fetal hemodynamic adaptive changes in IUGR (Verburg et al., 2008) wherein the standard assessment of cardiac function utilizes various biochemical and ultrasound indices in relation to disease severity (Crispi et al., 2008). Until now, very few studies have successfully utilized electrocardiographic parameters to elucidate ongoing fetal cardiac dysfunction.

Our study is based on the intimate relationship between the electrical conduction system and the overall structure of the heart. Since IUGR induced cardiovascular remodeling affects both cardiac structure and function, and electrocardiographic parameters are established indicators of cardiac function as well, a thorough investigation of fetal electrocardiography may help us understand the pathophysiology behind fetal growth restriction.

In 1906, Cremer inadvertently discovered transabdominal fetal electrocardiographic (FECG) extraction (Skillern et al., 2005). Many physicians since then have affirmed its potential clinical value. However, non-homogenous tissue conduction, insulating effects of the vernix caseosa, poor signal noise reduction and its inability to pinpoint the exact cardiac structural changes prevented its acceptance for clinical use. The progress in the development of abdominal FECG systems then lagged most especially after the development of transcervical extraction techniques that addressed the issues raised with the transabdominal route. The transcervical route through fetal ECG ST segment analysis (STAN) is currently being used in intrapartal monitoring. The S-T interval on ECG represents myocardial repolarization. Its use in conjunction with cardiotocography was found to significantly reduce the number of fetal scalp blood sampling, operative vaginal deliveries and Cesarean section for the indication of fetal distress. European and North American trials (Vayssiere et al., 2007) showed good correlation between STAN and fetal cord blood pH. One study reported that a significant ST event for screening pH < or = 7.15 (21.9%) was with sensitivity 38% (41/108), specificity 83% (252/303), PPV 45% (41/92) and NPV 79% (252/319), and for pH < or = 7.05, it was (3.4%), 62.5% (10/16), 79% (313/395), 11% (10/92), and 98% (313/319), respectively. In another study, a negative predictive value of 95.2% was achieved (Devoe et al., 2006). Other studies have found FECG useful in: (a) evaluating atrioventricular block (AVB) in Anti-RO positive pregnancies, (b) monitoring drug therapy, or (c) monitoring fetal anemia (Gardiner et al., 2007).

Recent advances in the technology of abdominal FECG addressed previous limitations thus prompting the reintroduction of transabdominal FECG (Sato et al., 2007). A novel system developed in Sendai, Japan was used in this study. The basic advantages of this system over other techniques were: (1) it was non-invasive, lead electrodes were applied on the maternal abdomen using an adhesive pad; (2) it eliminated interference from maternal cardiac signals and fetal movement thus producing clear ECG recordings; and (3) it compared well with the gold standard, magnetocardiography, in terms of gestational age-dependent ECG parameters, such as the QRS, PQ and QT intervals (Horigome et al., 2000). A minimum recording time of 20 min was usually needed from which the fetal heart rate could be detected in real time and online in more than 70% of patients. In about 30%, 5–30–min off line calculations were required. For this study, all results were calculated off line to ensure good resolution of signals. Moreover, the inclusion of simultaneous Doppler studies increased the resolution of the FECG extraction process.

Fetal ECG (FECG) has a voltage of 5–20 μV and signal amplitude of 1/50 of the maternal ECG (MECG) complex which averages 1,000 μV. For this study, the method used was blind source separation with reference signals (BSSR) wherein the FECG was isolated from the surrounding noise by bouncing the signal through various transfer points. From a mathematical point of view, FECG is derived from the following linear equations:

$$\begin{array}{l} \text{ChA }=\text{ a}1•\text{FECG}+\text{a}2•\text{MECG}+\text{noise}1\\ \text{ChB }=\text{ b}1•\text{FECG}+\text{b}2•\text{MECG}+\text{noise}2\\ \text{ChC }=\text{ c}1•\text{FECG}+\text{c}2•\text{MECG}+\text{noise}3\\ \text{where Ch }=\text{channel}\\ \text{FECG }=\text{fetal electrocardiography}\\ \text{MECG }=\text{ maternal electrocardiography} \end{array}$$

Measured signals like ChA, ChB, and ChC are mixed signals that include FECG, MECG and other sources of noise (Kimura et al., 2012). In the presence of large noise, reference signals, such as continuous Doppler recordings or adaptation of a lead ECG waveform allows for shape mimicry of FECG and for detection by imitating signal timing. Moreover, our method differs from other systems because it is non-invasive and mobile. It is non-invasive since it merely necessitates placement of adhesive electrodes on the maternal abdomen. In contrast ST analysis (STAN) (Ross et al., 2004), requires rupturing the amniotic membranes and transcervically attaching needles into the fetal scalp. Mobility is achieved using a simple ensemble of portable equipment, laptop and signal extractor, unlike other immobile complex systems, such as in fetal magnetocardiography (FMCG) (Leuthold et al., 1999; Kähler et al., 2002; Grimm et al., 2003).

The cardiac conduction system and the structural morphology of the heart are intimately related thus making fetal electrocardiography a possible means to assess alterations in cardiac structure and performance. Cardiac overload or increases in peripheral resistance (McDade et al., 2001; Mari et al., 2007; Romo et al., 2009; Bolin et al., 2016) are known to occur in cases of fetal growth restriction. The objective of this study was to assess whether abdominal FECG parameters can be developed as markers for evaluating the fetal cardiac status in IUGR.

## Materials and methods

In the Philippines and United Kingdom, all pregnant women who consulted or were admitted in the participating institutions, and were referred for fetal wellbeing studies, had met the inclusion criteria, and who did not have any of the exclusion criteria were included in the study (Table 1). The principal investigator obtained informed consent only after a thorough explanation and discussion of the study with the patient. Each patient then underwent a physical examination and interview prior to FECG recording and standard ultrasonographic and Doppler evaluation.

**Table 1:** Study criteria.

**INCLUSION CRITERIA**	**EXCLUSION CRITERIA**
Control group-singleton pregnancies from 20 weeks up to 33 weeks and 6 days of gestation with fetuses whose sonographic estimated fetal weight and abdominal circumference falls within the 10 to 90th percentile for gestational age. IUGR group-singleton pregnancies from 20 weeks up to 33 weeks and 6 days of gestation with fetuses whose sonographic estimated fetal weight and abdominal circumference falls below the 10th percentile for gestational age. IUGR staging will be based on the following Doppler findings: Stage 0 includes fetuses with normal umbilical artery and middle cerebral artery Doppler indices. Stage I includes fetuses with abnormal Doppler indices of the umbilical artery (increased resistance indices) or middle cerebral artery (cerebroplacental ratio of < 1). Stage II includes fetuses with absent or reversed Doppler flow of the umbilical artery. Stage III includes fetuses with absent or reversed Doppler flow of the a-wave of the ductus venosus.	The presence of any of the following: Chromosomal anomalies Congenital anomalies Premature labor Maternal medical contraindications to the use of electronic devices, such as pacemakers Maternal mental disability, coma or sensorial changes Severe maternal illness

FECG was recorded using a novel extraction method from composite abdominal signals involving cancellation of the maternal ECG and blind source separation with a reference signal (BSSR). The method of abdominal FECG was as follows (Kimura et al., 2006): A standardized placement of 14 adhesive electrodes was utilized:12 placed on the maternal abdomen, inclusive of one reference electrode; one placed at the right maternal thoracic position; and one placed on the maternal back. Bipolarly recorded data of channels were sampled every 1 ms at 1 kHz with a 16-bit resolution which was subjected to 1–100 Hz band-pass filtering. Simultaneous fetal cardiotocography was performed to identify the opening and closing of fetal cardiac valves.

Each FECG recording lasted 20 min. All recordings were anonymized and sent to Sendai, Japan for fetal ECG extraction. For each recording, an averaged ECG waveform was used as a reference signal for the BSSR algorithm to seek fetal QRST components in the orthogonal signals from the first and second channel after the BSSR. This is followed by fast non-linear state space projection (FNSSP) within the time domain to precisely separate noise from the fetal ECG. Also, the simultaneous Doppler signals measured represented the opening and closing of aortic valves and were used to distinguish the end of the T wave. The fetal ECG standard parameters evaluated included the following: QT, RR, QRS, ST, PR, and PQ intervals, height of P wave, QTc, PR/RR and heart rate. Raw data was returned to the principal investigator for statistical analysis. The correlation of these standard fetal ECG parameters with other ultrasound [amniotic fluid deepest pocket (AF DP) or index (AFI), head circumference to abdominal circumference ratio (HC/AC), femoral length to abdominal circumference ratio (FL/AC)] and Doppler markers [middle cerebral artery (MCA), umbilical artery (Umb A), uterine arteries (UA), and ductus venosus (DV)] were analyzed.

Data were collated and encoded in MSEXCEL 2013. Categorical data in the demographics, the maternal characteristics, and the diagnostic tests were described using frequency and percentages. Moreover, the continuous types of data were expressed in mean and standard deviation.

In comparing the mean and variances between control and FGR groups, the unpaired t-test was utilized to check the equality of variances as well as differences of means. Moreover, in testing associations among categorical types of data and groupings, the Chi Square test of independence and the 2 × 2 Fisher exact test were performed. Any associated *p* < 0.05 alpha were considered significant.

Furthermore, all the abdominal fetal electrocardiography parameters were tested for accuracy, such as sensitivity, specificity, negative, and positive predictive values. Any cut off values found to produce accuracy indices were presented.

The accuracy of computations was ensured through careful data processing and aided by IBMSPSS version 21 and NCSSPASS 2000 statistical software.

## Results

A total of 46 fetal ECG recordings were collected throughout the study period, 16 from the Philippines (RP) and 30 from the United Kingdom (UK). Out of the 46, only 20 Control and 15 IUGR fetuses were eligible after the final exclusion of patients with incomplete clinical data or poor ECG recordings.

Patients' demographics, maternal characteristics and clinical profiles were summarized in Tables 2–4. The mean age of patients was 30.86 ± 5.75 years (range: 17–40 years) where a majority (54%) of the study subjects were Asian (*p* = 0.018). All pregnancies (100%) were achieved through spontaneous conception in women with a mean gravidity score of 2.26 ± 1.52 and only 10 (28.6%) of these women reported a previous fetal loss. This evaluation revealed that there was a significant difference between the maternal weights of Control (C) and IUGR fetuses wherein mothers of the latter, on the average, weighed less (*p* = 0.034). Of all the indices of IUGR, HC/AC ratio alone was significant in predicting IUGR (*p* < 0.01). Moreover, for the IUGR sample population studied, there was an even distribution among the various stages of IUGR (11–17%) except in Stage III (3%).

**Table 2:** Comparison of patients' demographics between CONTROL and IUGR groups.

**Patients' demographics**	**CONTROL**		**IUGR**		**Total**		***p*-value**
	***n*** = **20**		***n*** = **15**		***n*** = **35**		
**MATERNAL AGE, YEARS**							
Mean(SD)	30.85	5.44	30.87	6.33	30.86	5.75	0.993
**ETHNICITY, n (%)**							
Asian	7	35%	12	80%	19	54%	0.018^*^
Black	5	25%	0	0%	5	14%	
White	8	40%	3	20%	11	31%	

p < 0.05 alpha

* *(significant)*.

*SD, standard deviation*.

**Table 3:** Comparison of maternal characteristics between CONTROL and IUGR groups.

**Maternal characteristics**	**CONTROL**		**IUGR**		**Total**		***p*-value**
	***n*** = **20**		***n*** = **15**		***n*** = **35**		
Spontaneous Conception, n (%)	20	100%	15	100%	35	100%	1.000
HT, mean(SD)	160.0	9.0	156.1	8.2	158.3	8.7	0.196
WT, mean(SD)	62.9	13.5	54.1	8.0	59.0	12.1	0.034^*^
BMI, mean(SD)	24.5	4.4	22.4	4.5	23.6	4.5	0.191
SMOKER, n (%)	0	0%	2	13%	2	6%	0.093
GA, mean(SD)	25.84	3.93	26.33	4.32	26.05	4.05	0.726
Gravidity, mean(SD)	2.25	1.55	2.27	1.53	2.26	1.52	0.975
Parity, mean(SD)	0.95	1.32	0.80	0.94	0.89	1.16	0.710

p < 0.05 alpha

* *(significant)*.

*HT, height; WT, weight; BMI, body mass index, GA, gestational age*.

**Table 4:** Comparison of clinical profiles between CONTROL and IUGR groups.

**Clinical profiles**	**CONTROL**		**IUGR**		**Total**		***p*-value**
	***n*** = **20**		***n*** = **15**		***n*** = **35**		
**EFW**, mean(SD)	926.80	490.21	657.20	442.21	811.26	482.84	0.103
**EFW parameters**, mean(SD)							
HC	233.58	38.43	219.79	41.78	227.67	39.90	0.319
AC	210.22	41.44	180.97	46.00	197.68	45.24	0.057
FL	46.33	9.32	39.38	11.12	43.35	10.56	0.053
HC/AC	1.12	0.06	1.24	0.11	1.17	0.10	<0.01^*^
FL/AC	0.22	0.01	0.22	0.01	0.22	0.01	0.301
GA	25.84	3.93	26.33	4.32	26.05	4.05	0.726
**Presentation, n (%)**							
Breech	6	30%	4	27%	10	29%	0.961
Cephalic	13	65%	10	67%	23	66%	
Transverse	1	5%	1	7%	2	6%	
**IUGR stage, n (%)**							
0	–	–	4	27%	4	11%	
1	–	–	4	27%	4	11%	
2	–	–	6	40%	6	17%	
3	–	–	1	7%	1	3%	

p < 0.05 alpha

* *(significant)*.

*EFW, estimated fetal weight; HC, head circumference; AC, abdominal circumference; FL, femoral length*.

Diagnostic Test Results (Table 5) showed significant differences between the C and IUGR groups. The most relevant changes were seen in the umbilical artery pulsatility index (Umb A PI), right and left uterine artery PI (UA PI) measurements and amniotic fluid deepest pool (AF DP) whose *p*-values were equal to 0.001, 0.001, < 0.01, and 0.040, respectively.

**Table 5:** Comparison of diagnostic test results between CONTROL & IUGR groups.

**Diagnostic test results**	**CONTROL**		**IUGR**		**Total**		***p*-value**
	***n*** = **20**		***n*** = **15**		***n*** = **35**		
**Umbilical artery PI**, mean(SD)	1.00	0.11	1.73	0.63	1.42	0.60	0.001^*^
**Umbilical artery EDF n (%)**							
Negative	0	0%	6	40.0%	6	17%	0.030^*^
Positive	11	55%	8	53.3%	19	54%	
Reversed	0	0%	1	6.7%	1	3%	
**Middle cerebral artery PI**, mean(SD)	1.54	0.26	1.49	0.34	1.50	0.31	0.698
**MCA Vmax**, mean(SD)	38.30	5.85	38.06	9.92	38.14	8.68	0.954
**MCA EDF, n (%)**							
Negative	13	65%	0	0%	13	37%	<0.01^*^
Positive	7	35%	15	100%	21	60%	
**CP RATIO**, mean(SD)	–	–	1.00	0.55	1.00	0.55	
**DV PI**, mean(SD)	0.45	0.08	0.74	0.40	0.67	0.37	0.180
**DV EDF n (%)**							
Negative	0	0%	1	7%	1	3%	0.582
Positive	4	20%	13	87%	17	49%	
**RT UT**, mean(SD)	0.85	0.19	1.74	0.79	1.26	0.70	0.001^*^
**EDN n (%)**							
Absent	13	65%	7	47%	20	57%	0.017^*^
Present	0	0%	4	27%	4	11%	
**LT UT**, mean(SD)	1.02	0.42	2.00	0.65	1.47	0.72	<0.01^*^
**EDN n (%)**							
Absent	12	60%	1	7%	13	37%	<0.01^*^
Present	1	5%	10	67%	11	31%	
**Amniotic fluid index**, mean(SD)	13.83	2.03	12.77	5.49	13.18	4.41	0.635
**Amniotic fluid deepest pool**, mean(SD)	5.09	0.95	3.95	1.45	4.41	1.37	0.040^*^
**Placental Grade n (%)**							
1	9	45%	7	47%	16	46%	0.922
2	11	55%	8	53%	19	54%	

p < 0.05 alpha

* *(significant)*.

*PI, pulsatility index; EDF, end diastolic flow; MCA, middle cerebral artery; Vmax, maximum velocity; CP, cerebroplacental ratio; DV, ductus venosus; RT UT, right uterine artery; LT UT, left uterine artery*.

**Figure 1:**
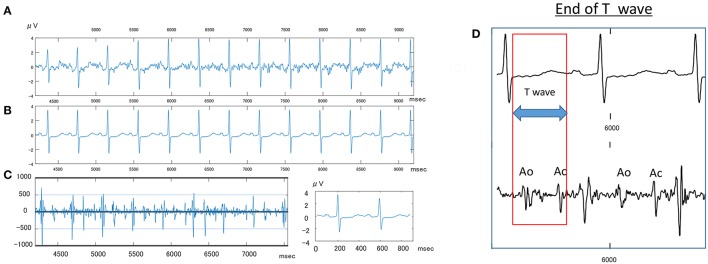
Sample fetal ECG recording in an IUGR fetus. **(A)** Shows the actual fetal ECG of an IUGR fetus at 34 weeks gestation. **(B)** Shows the averaged waveforms of the same interval. **(C)** Shows the Doppler wave signal recorded simultaneously by an attached continues Doppler transducer. **(D)** Shows how to measure the T wave, wherein Ao, opening of aortic valve; Ac, closing of Aortic valve.

Clear P-QRST complexes were recognized in all cases (Table 6, Figure 1). In the IUGR fetuses, both the QT and QTc parameters were significantly prolonged (*p* = 0.017 and *p* = 0.002, respectively). There was no correlation between significant ECG parameters and Doppler or other indices to predict IUGR (Tables 7, 8). A detailed table matrix of cut off values in detecting IUGR per fetal ECG parameter was generated and showed an inverse correlation, increasing sensitivities but decreasing specificities, with the prolongation of ECG parameters (Table 9).

**Table 6:** Comparison of fetal electrocardiographic parameters between CONTROL and IUGR groups.

**Abdominal fetal electrocardiography**	**CONTROL**		**IUGR**		**Total**		***p*-value**
	***n*** = **20**		***n*** = **15**		***n*** = **35**		
**Parameters**	**mean**	**±sd**	**mean**	**±sd**	**mean**	**±sd**	
QTc	0.37	0.04	0.40	0.03	0.38	0.04	0.017^*^
RR	403.13	24.48	414.20	20.02	407.87	23.04	0.163
P wave	49.93	9.99	50.39	11.15	50.13	10.35	0.897
PR	105.44	14.91	100.64	18.20	103.38	16.32	0.397
PQ	55.52	14.11	50.24	15.12	53.26	14.58	0.296
QRS	43.96	15.19	44.72	10.93	44.29	13.35	0.870
QT	234.29	22.68	255.82	12.72	243.51	21.70	0.002^*^
PR/RR	0.26	0.04	0.24	0.04	0.25	0.04	0.197
HR	149.34	8.68	145.17	6.96	147.55	8.15	0.137

p < 0.05 alpha

* *(significant)*.

*QTc-corrected QT interval; HR, heart rate*.

**Table 7:** Correlation of fetal electrocardiographic parameters and IUGR indices in the CONTROL group.

**Variables**	**Test statistics**	**QTc**	**RR**	**P wave**	**PR**	**PQ**	**QRS**	**QT**	**PR/RR**	**HR**
HC/AC	Pearson Correlation	−0.178	−0.442	−0.374	0.156	0.429	−0.261	−0.328	0.331	0.439
	*p*-value	0.454	0.051	0.105	0.511	0.059	0.266	0.158	0.154	0.053
FL/AC	Pearson Correlation	0.107	−0.144	0.046	−0.009	−0.042	0.044	0.072	0.048	0.152
	*p*-value	0.652	0.546	0.850	0.968	0.861	0.854	0.762	0.840	0.524
AFI	Pearson Correlation	−0.443	−0.506	−0.035	0.326	0.713	−0.042	−0.513	0.560	0.482
	*p*-value	0.319	0.246	0.940	0.476	0.072	0.929	0.239	0.191	0.274
AF DP	Pearson Correlation	−0.166	−0.0454	0.139	0.451	0.376	−0.357	−0.192	0.561	0.003
	*p*-value	0.648	0.901	0.702	0.191	0.285	0.312	0.595	0.092	0.992
Umb A PI	Pearson Correlation	−0.181	0.379	−0.193	−0.190	−0.031	0.021	−0.078	−0.405	−0.385
	*p*-value	0.593	0.250	0.569	0.575	0.928	0.951	0.820	0.217	0.243
MCA PI	Pearson Correlation	0.020	−0.414	−0.783	−0.463	0.212	−0.018	−0.205	−0.275	0.393
	*p*-value	0.966	0.356	0.037^*^	0.295	0.649	0.969	0.659	0.551	0.384
MCA Vmax	Pearson Correlation	−0.225	−0.208	−0.367	0.160	0.664	−0.385	−0.371	0.333	0.166
	*p*-value	0.628	0.654	0.418	0.732	0.104	0.393	0.412	0.465	0.723
DV PI	Pearson Correlation	0.332	0.722	−0.272	−0.084	0.296	0.315	0.463	−0.386	−0.740
	*p*-value	0.668	0.279	0.728	0.916	0.704	0.685	0.537	0.614	0.260

p < 0.05 alpha

* *(significant)*.

*AFI–amniotic fluid index; AF DP – amniotic fluid deepest pocket; Umb A–umbilical artery*.

**Table 8:** Correlation of fetal electrocardiographic parameters and IUGR indices in the IUGR group.

**Variables**	**Test statistics**	**QTc**	**RR**	**P wave**	**PR**	**PQ**	**QRS**	**QT**	**PR/RR**	**HR**
HC/AC	Pearson Correlation	0.142	−0.324	0.054	−0.272	−0.368	−0.420	0.020	−0.171	0.336
	*p*-value	0.613	0.240	0.8481	0.327	0.178	0.119	0.944	0.544	0.220
FL/AC	Pearson Correlation	−0.101	0.144	0.205	0.341	0.260	0.098	−0.062	0.279	−0.141
	*p*-value	0.721	0.608	0.464	0.213	0.345	0.729	0.825	0.313	0.617
AFI	Pearson Correlation	0.326	−0.495	0.335	0.002	−0.250	−0.0975	0.184	0.146	0.491
	*p*-value	0.328	0.122	0.313	0.995	0.458	0.776	0.588	0.669	0.125
AF DP	Pearson Correlation	0.155	−0.210	0.113	0.018	−0.062	0.023	0.101	0.110	0.210
	*p*-value	0.582	0.453	0.689	0.950	0.826	0.936	0.719	0.695	0.453
CP RATIO	Pearson Correlation	−0.248	0.120	0.0770	0.304	0.309	0.058	−0.262	0.255	−0.136
	*p*-value	0.373	0.672	0.785	0.270	0.262	0.836	0.345	0.359	0.628
Umb A PI	Pearson Correlation	0.391	−0.327	−0.285	−0.396	−0.266	−0.299	0.363	−0.288	0.324
	*p*-value	0.150	0.234	0.304	0.144	0.337	0.279	0.184	0.298	0.239
MCA PI	Pearson Correlation	−0.031	−0.240	0.071	0.276	0.280	−0.105	−0.142	0.323	0.214
	*p*-value	0.913	0.389	0.802	0.320	0.313	0.710	0.613	0.241	0.4447
MCA Vmax	Pearson Correlation	0.067	0.010	−0.078	0.157	0.247	−0.036	0.095	0.164	−0.014
	*p*-value	0.811	0.972	0.782	0.577	0.376	0.901	0.735	0.558	0.959
DV PI	Pearson Correlation	0.126	−0.096	−0.532	−0.566	−0.236	−0.417	0.122	−0.467	0.089
	*p*-value	0.682	0.754	0.061	0.044^*^	0.438	0.156	0.691	0.108	0.773

p < 0.05 alpha

* *(significant)*.

**Table 9:** Test of accuracy for the prediction of IUGR in cut off values of the different fetal electrocardiographic parameters.

**Parameters' tested cut off values**	**Sensitivity (%)**	**Specificity (%)**	**Likelihood ratio (+)**	**PPV (%)**	**NPV (%)**
**QTc**					
0.40	66.7	25.0	0.89	40.0	50.0
0.42	80.0	15.0	0.94	41.4	50.0
0.43	86.7	5.0	0.91	40.6	33.3
**RR**					
380.78	6.7	80.0	0.33	20.0	53.3
391.57	13.3	60.0	0.33	20.0	48.0
402.35	26.7	45.0	0.48	26.7	45.0
413.14	46.7	40.0	0.78	36.8	50.0
423.92	80.0	20.0	1.00	42.9	57.1
434.71	80.0	10.0	0.89	40.0	40.0
445.49	93.3	5.0	0.98	42.4	50.0
**P Wave**					
39.89	13.3	85.0	0.89	40.0	56.7
44.83	33.3	70.0	1.11	45.5	58.3
49.78	46.7	55.0	1.04	43.8	57.9
54.72	53.3	30.0	0.76	36.4	46.2
59.67	80.0	10.0	0.89	40.0	40.0
64.61	93.3	10.0	1.04	43.8	66.7
**PR**					
78.38	13.3	95.0	2.67	66.7	59.4
85.46	26.7	85.0	1.78	57.1	60.7
92.53	33.3	80.0	1.67	55.6	61.5
99.61	46.7	65.0	1.33	50.0	61.9
106.69	60.0	45.0	1.09	45.0	60.0
113.77	80.0	25.0	1.07	44.4	62.5
120.84	86.7	15.0	1.02	43.3	60.0
127.92	93.3	5.0	0.98	42.4	50.0
**PQ**					
49.17	53.3	70.0	1.78	57.1	66.7
55.73	66.7	45.0	1.21	47.6	64.3
62.3	73.3	30.0	1.05	44.0	60.0
68.87	93.3	25.0	1.24	48.3	83.3
75.43	93.3	5.0	0.98	42.4	50.0
**QRS**					
37.73	33.3	60.0	0.83	38.5	54.6
45.31	46.7	50.0	0.93	41.2	55.6
52.89	66.7	25.0	0.89	40.0	50.0
60.47	93.3	5.0	0.98	42.4	50.0
**QT**					
246.76	20.0	20.0	0.25	15.8	25.0
257.37	60.0	15.0	0.71	34.6	33.3
267.99	80.0	10.0	0.89	40.0	40.0
**PR/RR**					
0.21	33.3	90.0	3.33	71.4	64.3
0.24	46.7	75.0	1.87	58.3	65.2
0.26	53.3	50.0	1.07	44.4	58.8
0.28	80.0	30.0	1.14	46.2	66.7
0.3	93.3	15.0	1.10	45.2	75.0
0.32	93.3	5.0	0.98	42.4	50.0
**HR**					
135.95	13.3	90.0	1.33	50.0	58.1
139.7	20.0	90.0	2.00	60.0	60.0
143.44	26.7	75.0	1.07	44.4	57.7
147.18	73.3	60.0	1.83	57.9	75.0
150.93	73.3	55.0	1.63	55.0	73.3
154.67	93.3	30.0	1.33	50.0	85.7

## Discussion

Intrauterine growth restriction (IUGR) is defined as the failure of a fetus to reach its growth potential (Mari and Hanif, 2008). It is distinguished from fetuses that are merely small for gestational age (SGA), weighing below the 10th percentile, by signs of chronic hypoxia. It is a condition caused by several factors, alone or in combination, such as maternal disease, malnutrition, drugs and toxins, uteroplacental insufficiency, fetal aneuploidy, fetal abnormality, genomic imprinting, and congenital infections. This can be further classified as LATE (TERM or ASYMMETRIC) IUGR, which includes fetuses with onset of growth restriction at a gestational age ≥34 weeks or EARLY (PRETERM or SYMMETRIC) IUGR, wherein growth restriction occurs at <34 weeks gestation. This study focused on early IUGR fetuses for two reasons: first, the non-invasive fetal ECG system was best calibrated for fetuses between 16 and 32 weeks age of gestation and second, it was during this exact period, <34 weeks gestation, that the detection and evaluation of IUGR was most critical to reduce perinatal morbidity and mortality secondary to iatrogenic or indicated premature delivery.

IUGR is brought about by a persistent state of low oxygen or chronic hypoxia from increased placental impedance. This leads to the shunting of blood in the peripheral circulation toward more vital organs or the “Brain Sparing Effect.” Therefore, the fetal heart will undergo both physiologic and anatomic adaptation to meet the demands of increasing cardiac output. This constant cardiac remodeling eventually leads to both systolic and diastolic dysfunction where significant compensatory changes may be reflected in electrocardiographic as well as Doppler variations.

Overall, an increase in heart size due to free wall hypertrophy without ventricular dilatation causes progressive hemodynamic deterioration in IUGR fetuses (Bahtiyar and Copel, 2008). This is exemplified by a predictable pattern of worsening indices which progressively unfolds: umbilical artery pulsatility index increase, middle cerebral artery pulsatility index decrease, changes in right diastolic indices (right E/A, ductus venosus), changes in right systolic indices (right ventricular ejection force), then finally, both left diastolic and systolic cardiac dysfunction.

In clinical practice, Doppler indices in IUGR denote fetal cardiovascular status as well as the placental vascular conditions. Other standard measurements (AFI and the biometric parameters) on the other hand, are important signs of ongoing disease. The discovery of any significant correlation between ECG measurements and other IUGR markers, although not evident in this study, would allow a better understanding of the initiation and progression of cardiac structural change and its deterioration.

Our study findings show that fetal electrocardiography can distinguish between normal and IUGR fetuses as exhibited by the significant prolongation of QT and QTc intervals in the latter study group. Both these electrocardiographic parameters represent depolarization and repolarization of the ventricles and have been used as an indicator for deteriorating ventricular performance (Martin et al., 1994). These findings were consistent with earlier published data by Velayo et al evaluating abdominal fetal electrocardiography in congenital heart defects (CHD) in singleton pregnancies wherein certain cardiac pathologies exhibited particular ECG changes (Velayo et al., 2011). For example, PR and QTc intervals were prolonged in fetuses with dilated cardiomyopathy (DCM). They concluded that FECG had a role in the analyses of fetal cardiac pathophysiology not only in congenital heart disease but also in acquired cardiac maladaptive geometry which is the case in IUGR (Saba et al., 2005). The latter also supports the results of studies on twin to twin transfusion syndrome where progression of disease and simultaneous fetal cardiac changes can be evaluated. The same research group in collaboration with a center in the United Kingdom published an evaluation of fetal cardiac performance in twin-to-twin transfusion syndrome wherein the cardiovascular status of the donor and recipient twins differed based on fetal Doppler studies (Velayo et al., 2012). Again, the QT interval and QTc were significantly prolonged this time in the donor fetus. This fact was interesting since compared to its enlarging recipient counterpart, the evolution of a donor twin has been likened to a fetus with IUGR.

Studies using FMCG (Leuthold et al., 1999; Kähler et al., 2002; Grimm et al., 2003; Bolin et al., 2016) have shown that in the normal fetus ECG parameters lengthen as gestational age increases. The prevailing theory for this is that prolongation is caused by the increase in size of the fetal heart. Moreover, in FMCG studies involving IUGR fetuses, P wave, PR and QRS were significantly shorter, making the abbreviation of parameters signs of cardiac abnormality (Grimm et al., 2003; Bolin et al., 2016). Our findings using an abdominal FECG system agree with the first conclusion, where the lengthening of intervals with age is dependent on the cardiac mass. But our results of prolonged ECG parameters in this study and in earlier reports by our group, are inconsistent with FMCG findings (Velayo et al., 2011, 2012). A possible explanation for the prolongation in parameters is that in IUGR fetuses, the cardiac size increases in relation to fetal size, part of the brain sparing effect.

Interestingly, recent reports involving other non-invasive abdominal FECG systems have shown promising results for the use of phase-rectified signal averaging measurements (PRSA) and fetal heart rate variability (FHRV) in IUGR fetuses (Stampalija et al., 2015a,b). PRSA signals are representative of the current maturation status of the fetal autonomic nervous system (ANS) where lower acceleration capacity (AC) and deceleration capacity (DC) signals have been observed in IUGR fetuses with or without brain sparing. One study found a significant association between either AC and DC and the middle cerebral artery pulsatility index (PI; *p* = 0.01; *p* = 0.005) (Stampalija et al., 2015a). With FHRV, short term variability (STV) evaluation is known to reflect the status of the fetal oxygen supply, therefore indicating the fetal acid-base status. STV was seen to shorten in cases of IUGR (Stampalija et al., 2015b). All these parameters can identify the different behaviors of the heart rate and will give investigators a panoramic view of electrocardiopathology.

In future investigations, we propose the inclusion of parameters, such as the cardiac circumference (CC) to thoracic circumference (TC) or CC/TC ratio to elucidate the FECG phenomenon of cardiac size dependence. Furthermore, a comprehensive study that includes fetal ECG, Doppler and phase-rectified signal averaging measurements would potentially elucidate a more accurate cardiophysiologic model of IUGR.

Cardiac remodeling in IUGR is at least a three-dimensional process where anatomical changes cannot be considered alone but synchronously as a function of time and fetal size as well. In unaffected fetuses, the range of normal HR for instance varies with gestational age and can be observed as changing with developing maturity. This is because fetal heart structure and function continues to develop all throughout gestation and in the first year after delivery. This elasticity or penchant for adaptation is no longer a property of adult human hearts and is crucial in the configuration of diagnostic models in the fetus. For IUGR fetuses, setting cut off values for predictive markers will have to take multivariate algorithms into consideration. The cut off values for each ECG parameter in this study showed that fetal ECG is sensitive in detecting ongoing fetal cardiac remodeling but not a specific tool as it cannot be used to distinguish the cause of cardiac change, i.e., it cannot be used to distinguish between IUGR, congenital anomalies or even twin-to-twin transfusion syndrome. The potential for fetal ECG as a specific diagnostic tool lies in developing its vector analysis capability for determining specific heart structures involved (e.g., inferior heart wall, left ventricle or ventricular septum).

Detection of the subtle signs of cardiovascular remodeling is critical to monitoring hypertension and hypervolemia experienced by IUGR fetuses. The availability of noninvasive fetal ECG technology has ushered in the newest frontier of fetal cardiac studies to address this. It will increase our knowledge of this little-known area of electrocardiophysiology and ultimately, benefit our smallest of patients.

## Ethics statement

This research conformed with appropriate Good Clinical Practice (GCP) Guidelines. The protocol was approved by the respective research ethics boards of both the University of the Philippines-Manila [UPMREB (OBG) 2015-065-01] and the Harris Birthright Research Centre, Kings College, London (REC 02-03-033) followed by memorandums of agreement for collaborative research drawn between these centers prior to commencement of the study. All subjects gave written informed consent in accordance with the Declaration of Helsinki.

## Author contributions

CV designed the study; all authors substantially contributed to the experiments; CV and KN handled all patient recruitment and care in London; CV and JS handled all patient recruitment and care in Manila. YK and KF extracted and analyzed the fetal ECG at Tohoku University; CV wrote the manuscript with contributions from all authors.

### Conflict of interest statement

The authors declare that the research was conducted in the absence of any commercial or financial relationships that could be construed as a potential conflict of interest.
